# Continuous cryotherapy vs. traditional cryotherapy after total knee arthroplasty: A systematic review and meta-analysis of randomized controlled trials

**DOI:** 10.3389/fsurg.2022.1073288

**Published:** 2023-01-11

**Authors:** Meng-Meng Liu, Mian Tian, Changqi Luo, Shicheng Wang, Long Shao

**Affiliations:** ^1^ Department of Pathology, Huzhou Central Hospital, Affiliated Central Hospital Huzhou University, Huzhou, China; ^2^ Department of Orthopaedic Surgery, Dianjiang People’s Hospital of Chongqing, Chongqing, China; ^3^ Department of Orthopaedic Surgery, The Second People’s Hospital of Yibin, Yibin, China; ^4^ Department of Orthopaedic Surgery, Ningbo No.6 Hospital, Ningbo, China

**Keywords:** cryotherapy, total knee arthroplasty, postoperative pain, analgesics consumption, swelling, range of motion, cost

## Abstract

**Background:**

Cryotherapy is widely applied to relieve pain and improve functional outcomes after total knee arthroplasty (TKA). New cryotherapy devices have recently been developed to guarantee a fixed temperature for a prolonged time. Therefore, we conducted a systematic review and meta-analysis to compare continuous cryotherapy and traditional cryotherapy (ice bag or gel pack) for patients after TKA.

**Methods:**

This study was conducted according to a predefined protocol registered on PROSPERO. Two independent reviewers performed an electronic database search of PubMed, Embase, Cochrane, Web of Science, Google Scholar, and ClinicalTrials.gov. Dichotomous outcomes were reported as risk difference (RD) with 95% confidence intervals (CIs), and continuous outcomes were reported as mean difference (MD), or standardized mean difference (SMD) with 95% CIs.

**Results:**

Seven trials enrolling a total of 519 patients were included. There were no differences in pain intensity (MD: −0.54, 95% CI: −1.55 to 0.47; *P* = 0.30), analgesics consumption (MD: −0.37, 95% CI: −1.28 to 0.55; *P* = 0.43), postoperative range of motion (MD: 0.47, 95% CI: −4.09 to 5.03; *P* = 0.84), swelling of the knee joint, blood loss, change in hemoglobin, or transfusion rate. Meanwhile, there were no differences in length of hospital stay (MD: −0.77, 95% CI: −1.62 to 0.08; *P* = 0.07) and adverse events (RD: 0, 95% CI: −0.02 to 0.03; *P* = 0.74). In addition, continuous cryotherapy leads to extra costs and resources than traditional cryotherapy.

**Conclusions:**

Continuous cryotherapy does not appear to offer significant benefits for TKA when compared with traditional cryotherapy. Based on currently available evidence, traditional cryotherapy is still recommended as continuous cryotherapy is not cost-effective. Further well-designed studies with larger sample sizes are warranted to further confirm these preliminary results.

**PROSPERO Registration:** Identifier [CRD42022308217].

## 1. Introduction

Total knee arthroplasty (TKA) is an effective surgical intervention for end-stage arthritis of the knee joint, which could provide better overall improvements in function, mobility, pain, and health-related quality of life (1, 2). Despite several studies with short- to mid-term follow-up have reported excellent results with high rates of satisfaction, the postoperative period after TKA may be pretty challenging: patients may experience acute pain, potential blood loss, local swelling, and edema resulting from tissue damage and acute inflammatory responses, restricted motion, and stiffness of the knee joint, reduced quadriceps strength, and finally lead to delayed recovery and prolonged hospital stay (3–5). Thus, even with the latest advances in multimodal pain management protocols, surgical and anesthetic techniques, TKA remains a difficult procedure for most patients. It is, therefore, a pressing need for the introduction and implementation of the enhanced recovery after surgery (ERAS) principles, which aim to optimize perioperative care, reduce complications, shorten the length of hospital stay, and reduce readmission rates and costs (6–8). Cryotherapy, as a nonpharmaceutical treatment, plays a vital role in addressing immediate postoperative complications, mainly for severe pain and significant swelling (9, 10).

Cryotherapy, also known as cold therapy, was utilized for inflammation and infection treatment as early as 3,000 BC, and was utilized for anesthesia before operations and amputations for its analgesic and numbing effects in the 1800s (11, 12). At present, cryotherapy is still commonly recommended and widely applicated following orthopaedic procedures, which is also utilized to enhance recovery and outcomes after TKA (13). Despite many advances in postoperative rehabilitation, cryotherapy remains popular and is universally considered appealing for its minimal disadvantages compared with the possible benefits. External application of cryotherapy in TKA is the application of external cold mediums to the skin around the knee joint and is supposed to reduce the intra-articular temperature, which on the one hand, could slow the conduction velocity of nerve fibers and potentially reduce pain transmission, and on the other hand, could reduce peripheral blood flow due to circulating vasoconstriction and therefore decrease the local inflammation and swelling (13). Traditionally, ice bag or gel pack is the most common and economical cryotherapy method, which is typically discontinuous with unregulated cold temperature and demands a manual replacement by the staff nurses (14). Therefore, continuous cryotherapy devices have been developed to deliver a steady cooling temperature for a prolonged time (15). However, it remains unclear whether the newly developed continuous cryotherapy devices were superior to traditional ice/gel pack for TKA.

A broad scope of the literature has suggested that the volume of randomized controlled trials (RCTs) specifically focusing on continuous cryotherapy vs. traditional cryotherapy has increased, and findings are conflicting (16–22). The aim of this study was to perform a comprehensive systematic review and use a meta-analytic approach to pool outcomes to compare the efficacy, safety, and cost-effectiveness of continuous cryotherapy to traditional cryotherapy for TKA.

## 2. Methods

The present systematic review and meta-analysis was designed in accordance with the guidelines proposed by the Cochrane Collaboration in the Cochrane Handbook for Systematic Reviews of Interventions (http://www.cochrane-handbook.org) and completed according to a predefined protocol, which has been listed on the International Prospective Register of Systematic Reviews (PROSPERO; registration number CRD42022308217) (23). The study was completed in adherence with the PRISMA (Preferred Reporting Items for Systematic Reviews and Meta-Analyses) statement (24).

### 2.1 Literature search

We searched the following electronic bibliographic databases from inception to March 2,022 to capture all recent relevant studies: PubMed, Embase, The Cochrane Library (Cochrane Database of Systematic Reviews), Web of Science, and Google Scholar,. We performed electronic searches using exploded Medical Subject Headings (MeSH) terms with corresponding keywords. The search was broad and applied no language restriction. A detailed description can be found in Appendix 1. In addition, we further searched the ClinicalTrials.gov registry (https://clinicaltrials.gov/) and checked the reference lists of all included full-text articles and previous systematic reviews to identify any additional eligible studies. Corresponding authors of included articles were contacted, where possible, to obtain detailed information or numerical data.

### 2.2 Study eligibility and selection

Two investigators independently conducted the initial electronic databases search and carefully reviewed all yielded records for inclusion using pre-determined eligibility criteria. All records were screened by title, abstract, and keywords for possible inclusion, and subsequently, identified as “included”, “excluded”, or “required further retrieval” to identify eligibility. No language or publication database filter was applied. Any discrepancies were resolved through discussion by the review team.

The inclusion criteria were:

(i) Population: adult patients undergoing TKA;
(ii) Intervention: received continuous cryotherapy (without compression) after TKA;
(iii) Comparison: received traditional cryotherapy after TKA;
(iv) Outcomes: reporting at least one of the outcomes of interest listed below;
(v) Study type: RCT.

Exclusion criteria were non-RCT interventional studies, observational studies, conference abstracts, editorials, correspondence, expert opinions, case series or reports, and unavailable full texts.

### 2.3 Data review and extraction

Two independent reviewers extracted details pertaining to the participants from each included trial. The following data were extracted from each included study: first author; year of publication; study location; publication journal; study design; clinical settings; study population; demographic data; intervention management; control management; outcomes of interest. These extracted data were entered into a standardized data extraction form. When the information was unclear or missing, we attempted to contact the corresponding authors of the original studies. The differences in the extracted data were discussed and resolved by referring to the original article by the panel of all the reviewers. The main outcomes of interest were the efficacy, safety, and cost-effectiveness of continuous cryotherapy when compared with traditional cryotherapy. In detail, the primary outcomes include pain intensity, analgesics consumption, postoperative range of motion (PROM), and swelling of the knee joint; while the secondary outcomes include blood loss, change in hemoglobin, transfusion rate, adverse events, length of hospital stay, and cryotherapy costs.

### 2.4 Quality assessment

Two reviewers independently evaluated the risk of bias of each study using the assessment tool from the Cochrane Handbook (25). The major domains of bias (random sequence generation, allocation concealment, blinding of participants and personnel, blinding of outcome assessment, incomplete outcome data, selective reporting, and other bias) in each trial were reviewed. Each study was graded as “low risk of bias”, “unclear risk of bias”, or “high risk of bias”. The highest risk score from any one domain was used to inform the overall risk. If the highest risk score was “unclear risk of bias” but occurred across multiple domains, it was classed as high risk of bias. Therefore, to be of low risk of bias overall, the trial had to be at low risk of bias across all domains. The disagreements between the two reviewers were resolved *via* discussion and consensus.

### 2.5 Statistical analysis

This meta-analysis was performed using Review Manager version 5.3 (Nordic Cochrane Center) for all prespecified outcomes if three or more studies reported the outcome (23). The risk differences (RDs) with 95% confidence intervals (CIs) were calculated for dichotomous data; and the mean differences (MDs) or standardized mean difference (SMD) with 95% CIs were calculated for continuous data. When the mean values are not available for continuous outcomes, the median values was utilized for estimation; other potential missing data will be estimated using the methods described in the Cochrane Handbook (23). A random-effects model was used due to anticipated heterogeneity. Results were reported in a Forest plot with 95% CIs. Heterogeneity was assessed *via* three means: visual inspection of overlapping confidence intervals, the statistical heterogeneity across studies quantified using *I*^2^ statistics, with *P* ≥ 0.05 considered statistically non-significant. Heterogeneity will be considered to be substantial if the *I*^2^ value > 50%. All *P* values were two-sided, and a *P* value < 0.05 was considered to be statistically significant evidence.

## 3. Results

### 3.1 Study selection

**Figure 1:**
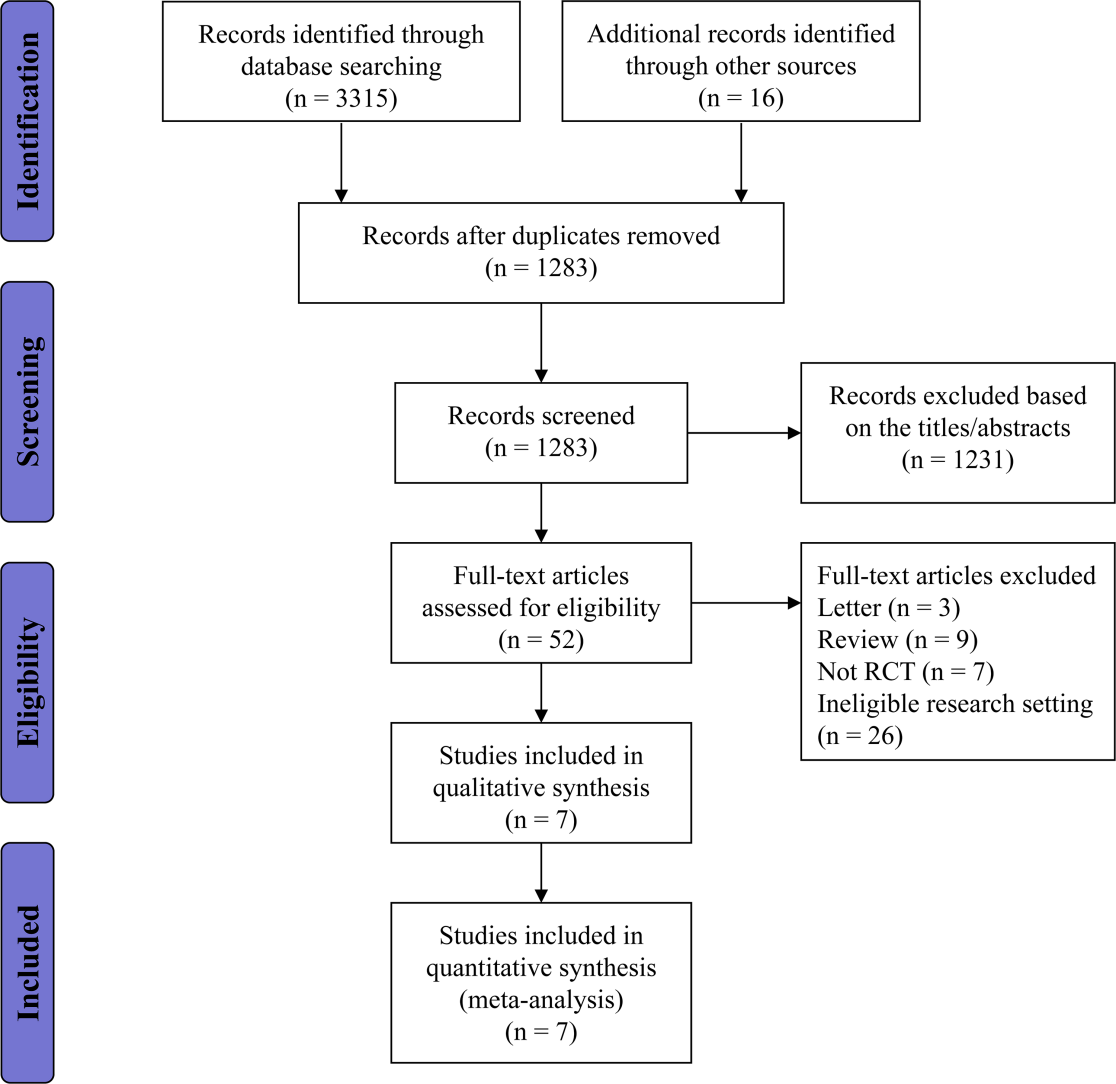
Study flow diagram showing the number of records identified and excluded at each stage.

In total, 1,387 articles were obtained from the electronic search strategy, with an additional 16 articles identiﬁed through other resources. After the removal of duplicates and irrelevant references, 35 publications were thought to be potentially eligible for inclusion and further assessed for eligibility. Overall we excluded 28 publications for not meeting the inclusion criteria, and seven RCTs were included. The flow diagram with the number of and reasons for exclusions at each stage is provided in Figure 1.

### 3.2 Characteristics of included studies

The characteristics of included studies can be found in the study characteristics tables (Tables 1, 2). Seven trials were included in our meta-analysis, which randomized 519 patients into continuous cryotherapy group (*n* = 263) and traditional cryotherapy (*n* = 256). These studies were published between 2012 and 2019, with a sample size ranging from 44 to 100. Notably, the application protocols between the continuous and traditional cryotherapy groups differed significantly with respect to the applied time and intervals, and the difference is even more significant among studies.

**Table 1:** Baseline characteristics of the included studies.

Author	Year	Region	Journal	Study Dates	Sample size
Demoulin (16)	2012	Belgium	Annals of Physical and Rehabilitation Medicine	Not reported	44
Thienpont (17)	2014	Belgium	Clinical Orthopaedics and Related Research	January 2012–October 2012	100
Bech (18)	2015	Canada	Physiotherapy Canada	February 2009–May 2012	71
Schinsky (19)	2016	US	Orthopaedic Nursing	June 2012–September 2013	97
Ruffilli (20)	2017	Italy	Journal of Knee Surgery	2013–2014	50
Sadoghi (21)	2018	Austria	International Orthopaedics	December 2011–April 2013	97
Karaduman (22)	2019	Turkey	Medicina (Kaunas)	January 2015–January 2016	60

**Table 2:** Baseline characteristics of the included population.

Author	Group size	Groups	Cold treatment	Device	Tourniquet use	Outcomes	Follow-up
Demoulin et al. 2012 (16)	22	Cryotherapy device	Applied for 20 min, five times a day. From POD 2 to discharge, except the weekend.	Aircast Cryocuff combined with AutoChill System (Aircast, Inc., Summit, New Jersey)	Used for 45–55 min	Pain intensity; swelling of the phatologic knee; ROM; cutaneous temperature of the knee	Not reported
	22	Ice/gel pack	Applied for 20 min, five times a day. From POD 2 to discharge, except the weekend.				
Thienpont et al. 2014 (17)	50	Cryotherapy device	The cTreatment device was used immediately after surgery for 4 h of continuous cooling at 11°C (range, 6–15°C). The day after surgery, the device was applied for 2 h after standard physiotherapy and repeated in the afternoon. During the evening and night, patients were allowed to continuously used during the night.	cTreatment system (Waegener, Beerse, Belgium)	Used for 55 ± 9 min	VAS score; analgesics consumption; ROM; swelling of the knee; blood loss	6 weeks for data collection and 3 months for adverse events
	50	Ice/gel pack	Patients received 15 min of cold pack (conserved at –17°C) treatment on arrival to the recovery room and again on arrival to the ward, and repeated 2 h and 4 h after surgery. The following days patients received cold pack cryotherapy 15 min after their physiotherapy session (11 AM and 3 PM) and during the evening and night whenever they considered it useful for comfort and pain control.				
Bech et al. 2015 (18)	37	Cryotherapy device	The device was applied immediately after surgery and remained in place for 48 h, except for brief periods: after 1 h, and every 4 h thereafter, for nursing assessment for skin or nerve damage; during exercise; and during ambulation.	DonJoy Iceman (DJO Canada, Mississauga, ON)	Not reported	Pain intensity; ROM, nausea or vomiting, opioid use, blood loss, lower limb function, hospital length of stay, patient-reported compliance and satisfaction	6 weeks after TKA
	34	Ice/gel pack	The operated limb was wrapped with an elastic bandage for 48 h after surgery to help control the degree of compression between groups. In the PACU and on the unit, the control group received ice bags at a frequency requested by the patient (usual care) for 48 h.				
Schinsky et al. 2016 (19)	49	Cryotherapy device	POD 1–3 continuous; POD4-10 1 h on and 1 h off while awake and continuous while asleep; POD11 as needed for pain control, continuous for 1-hour intervals; not to exceed 12 h/day while awake; may be used continuously as needed for pain control while asleep.	Polar Care Glacier (Breg, Inc.)	Used	Analgesics consumption; Length of stay; drain output; change in hemoglobin; change in hematocrit; allogeneic blood transfusions; ROM.	3 weeks and 6 weeks after TKA
	48	Ice/gel pack	POD 1–3 continuous; POD4-10 1 h on and 1 h off while awake and continuous while asleep; POD11 as needed for pain control: continuous for 1-hour intervals; not to exceed 12 h/day while awake; may be used continuously as needed for pain control while asleep. (replace the gel packs with fresh, frozen packs every 3–4 h)				
Ruffilli et al. 2017 (20)	24	Cryotherapy device	Hilotherm device was applied in the operating room and the device was turned on in the ward. The day after surgery, the elasto-compressive bandage was removed and the Hilotherm device was applied over the skin.	Hilotherm (Hilotherm GmbH, Germany)	Used for 92.5 ± 15.2 min and 93.8 ± 16.7 min, respectively	Edema reduction; blood loss, transfusion requirement, pain, ROM	1 week after TKA
	26	Ice/gel pack	Ice cold packs were applied over the elasto-compressive bandage, and changed every 30 min. The day after surgery, the elasto-compressive bandage was removed and the ice packs was applied over the skin.				
Sadoghi et al. 2018 (21)	51	Cryotherapy device	The device was applied immediately after TKA in the postanaesthesia care unit for six hours in total, applied each day for four hours in total, two hours in the morning and two hours in the afternoon.	cTreatment system (Waegener, Beerse, Belgium)	Not reported	Pain intensity; ROM; analgesics consumption; swelling of the knee; hospitalisation length; adverse effect	Not reported
	46	Ice/gel pack	Cold packs were applied three times per day for 20 min each throughout the whole trial.				
Karaduman et al. 2019 (22)	30	Cryotherapy device	Cryotherapy was applied for the ﬁrst 6 h after TKA; On POD1, it was applied at 2 h intervals. On POD2–3, it was applied every 6 h for 2 h.	cTreatment system (Waegener, Beerse, Belgium)	Used	Hemoglobin levels; VAS pain scores; analgesic requirement; ROM; swelling of the knee; length of hospital stay	Monitored for 12–24 months after TKA
	30	Ice/gel pack	A cold pack (gel ice) was applied as standard treatment for 20 min every 2 h for 3 days postoperatively.				

PACU, post-anesthesia care unit; POD, postoperative day; ROM, range of motion; VAS, visual analog scale.

### 3.3 Risk of bias in included studies

**Figure 2:**
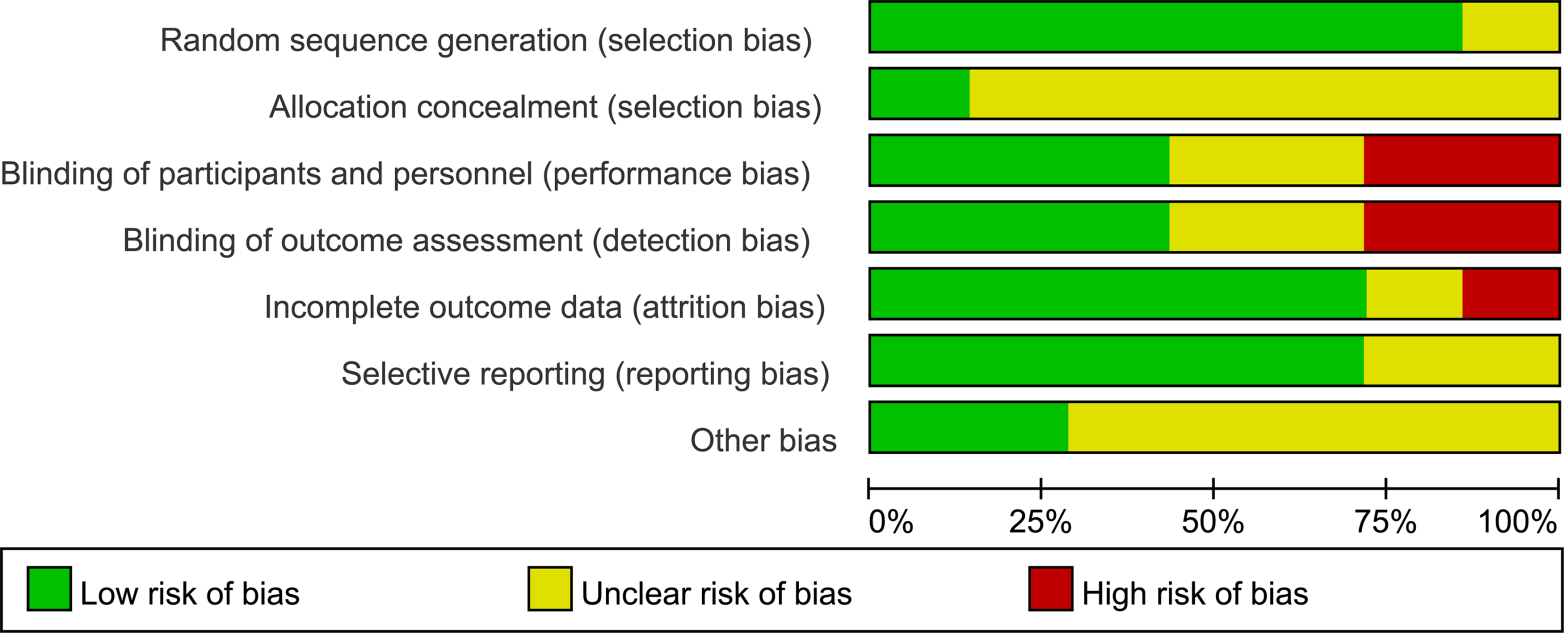
Summary of risks of bias of included studies.

**Figure 3:**
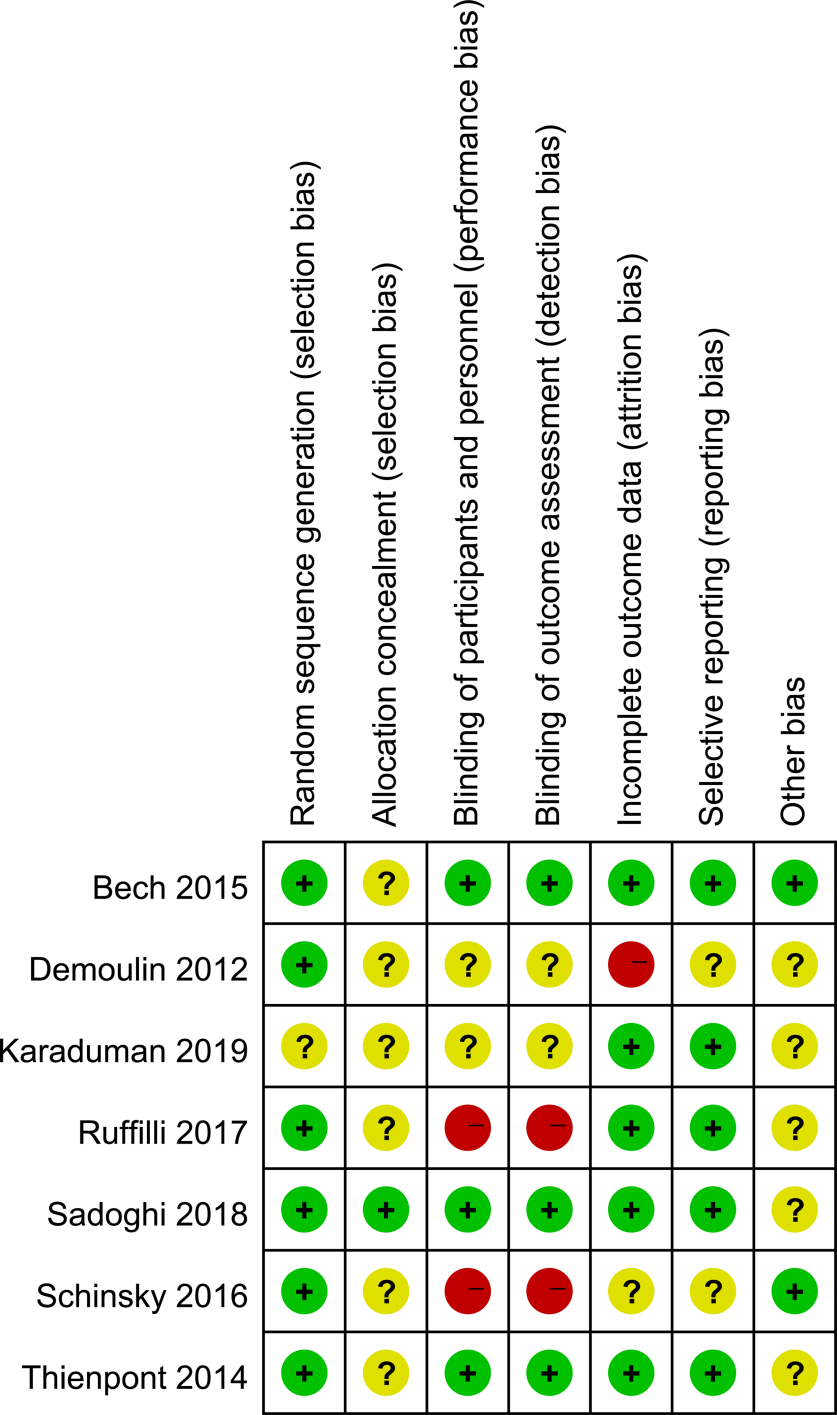
Risk of bias in individual studies.

No trials were considered to be at low risk of bias. Three studies were judged to be at high risk of bias, and four studies were felt to be at unclear risk of bias (Figures 2, 3). More specifically, adequate randomized sequence generation was reported in six trials, while appropriate allocation concealment was conducted in one trial. Blinding of outcome assessments was achieved by three trials, thus, the primary efficacy outcome and other outcomes assessment may have been affected by lack of blinding to some extent.

## 4. Outcomes

### 4.1 Primary outcomes

#### 4.1.1 Pain

**Figure 4:**
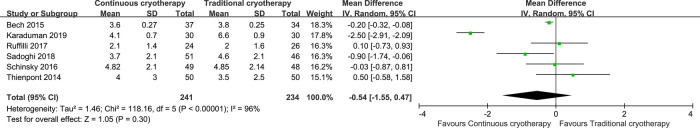
Forest plot for pain intensity.

**Figure 5:**
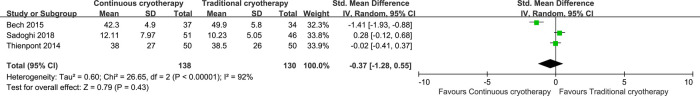
Forest plot for analgesics consumption.

Data for pain intensity were reported by six trials that recruited 475 patients (*n* = 241 vs. *n* = 234 in the continuous cryotherapy group and traditional cryotherapy group, respectively). Meta-analysis was performed on studies that reported a pain score for participants at 48 h postoperatively (Figure 4). There was no statistically significant difference in the pain score at 48 h between the continuous cryotherapy group and the traditional cryotherapy group (MD: −0.54, 95% CI: −1.55 to 0.47; *P* = 0.30). A high level of heterogeneity was observed (*I*^2^ = 96%). Data for analgesics consumption were reported by three trials that recruited 268 patients (*n* = 138 vs. *n* = 130 in the continuous cryotherapy group and traditional cryotherapy group, respectively). SMD was used as there were differences in the calculating conversations of analgesics consumption. There was no statistically significant difference in analgesics consumption between the continuous cryotherapy group and the traditional cryotherapy group (SMD: −0.37, 95% CI: −1.28 to 0.55; *P* = 0.43) (Figure 5). A high level of heterogeneity was observed (*I*^2^ = 92%).

#### 4.1.2 Swelling

**Figure 6:**
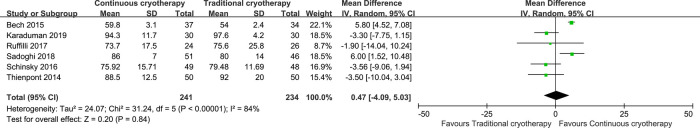
Forest plot for postoperative range of motion.

Data for PROM were reported by six trials that recruited 475 patients (*n* = 241 vs. *n* = 234 in the continuous cryotherapy group and traditional cryotherapy group, respectively). There was no statistically significant difference in the PROM between the continuous cryotherapy group and the traditional cryotherapy group (MD: 0.47, 95% CI: −4.09 to 5.03; *P* = 0.84) (Figure 6). A high level of heterogeneity was observed (*I*^2^ = 84%).

Knee circumference is another parameter that reflects swelling of the knee joint and was reported in three trials. Meta-analysis was not performed as two trials reported the postoperative knee circumference while one trial reported the difference in knee circumference. Overall, all three trials found no statistically significant difference in knee circumference between the continuous cryotherapy group and the traditional cryotherapy group.

### 4.2 Secondary outcomes

#### 4.2.1 Blood loss

Only one study reported blood loss and there was no statistically significant difference between the continuous cryotherapy group and the traditional cryotherapy group.

#### 4.2.2 Change in hemoglobin

Three trials reported data for hemoglobin changes, and meta-analysis was not performed because two trials reported the preoperative and postoperative hemoglobin while one trial reported the change in hemoglobin. Only one study detected a statistically significant difference between the continuous cryotherapy group and the traditional cryotherapy group (22).

#### 4.2.3 Transfusion rate

Data for transfusion rate was reported by three trials, and meta-analysis was not performed as two trials reported the number of transfusions while one study reported the number of units of allogeneic blood transfusions. Overall, all three trials found no statistically significant difference in transfusion rate between the continuous cryotherapy group and the traditional cryotherapy group.

#### 4.2.4 Length of hospital stay

**Figure 7:**
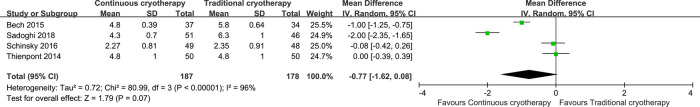
Forest plot for the length of hospital stay.

Data for the length of hospital stay were reported by four trials that recruited 265 patients (*n* = 187 vs. *n* = 178 in the continuous cryotherapy group and traditional cryotherapy group, respectively). There was no statistically significant difference in the length of hospital stay between the continuous cryotherapy group and the traditional cryotherapy group (MD: −0.77, 95% CI: −1.62 to 0.08; *P* = 0.07) (Figure 7). A high level of heterogeneity was observed (*I*^2^ = 84%).

#### 4.2.4 Safety

**Figure 8:**
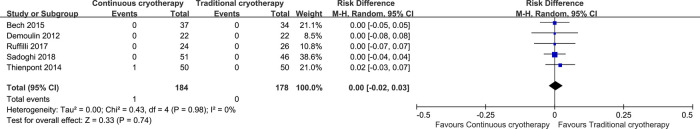
Forest plot for adverse events.

Data for adverse events were reported by six trials that recruited 362 patients (*n* = 184 vs. *n* = 178 in the continuous cryotherapy group and traditional cryotherapy group, respectively). There was no statistically significant difference in the incidence of adverse events between the continuous cryotherapy group and the traditional cryotherapy group (RD: 0, 95% CI: −0.02 to 0.03; *P* = 0.74) (Figure 8).

#### 4.2.4 Cost

Data for cryotherapy costs were reported by three trials. Thienpont et al*.* reported that the cost of continuous cryotherapy is $ 520, Schinsky et al*.* reported that continuous cryotherapy costs $97.34 than traditional cryotherapy per patient, while Karaduman reported that they found no significant additional costs associated with the use of continuous cryotherapy.

## 5. Discussion

### 5.1 Main findings

Our meta-analysis comprehensively and systematically reviewed the currently available literature, and the study results suggest that continuous cryotherapy does not appear to offer significant clinical benefits for TKA compared with traditional cryotherapy. There were no significant differences in pain intensity, analgesics consumption, postoperative range of motion, swelling of the knee joint, blood loss, change in hemoglobin, transfusion rate, length of hospital stay, and adverse events. In addition, continuous cryotherapy may lead to extra costs and resources than traditional cryotherapy.

### 5.2 Implication for clinical practice

Although TKA shows long-lasting clinical and structural improvement for the management of severe osteoarthritis, patients in the immediate postoperative period are often associated with acute pain, hidden bleeding, severe edema, and reduced range of motion. Cryotherapy has been shown to appreciably reduce the intraarticular temperature, especially in the knee, blood flow by vasoconstriction, the local inﬂammatory reaction, postoperative bleeding and swelling, pain transmission, and the length of hospital stay (26–29). In the clinic, several cryotherapy options are available, including: (i) the first-generation cold therapy such as ice bag or gel pack; (ii) second-generation cryotherapy devices with circulating ice water with or without compression; (iii) third-generation devices with advanced computer-assisted devices to provide continuous controlled cold therapy (17). Compared with ice/gel pack, advanced cryotherapy devices are developed and are expected to be even more efficient as they maintain a steady low temperature for an extended time. Thus, in theory, continuous cryotherapy could play a better role in fast-track rehabilitation after TKA by reducing inflammation, pain, and swelling. However, this meta-analysis observed no differences in clinical outcomes between continuous cryotherapy and traditional cryotherapy, which could be caused by several factors such as the level of tissue penetration of cold therapy, method of cryotherapy, time of application, and types of outcome measurement. TKA-induced inflammation leads to a significant increase in temperature deep inside the knee joint, and the effect of cryotherapy after TKA is closely related to the temperature-dependent mechanism (13). After the cold temperature penetrates tissues and reaches the intended area, which reduces inflammation, reduces nerve conduction velocity, induces local vasoconstriction, and reduces blood flow to muscles (30–35). Although continuous cryotherapy is a more effective treatment to consistently maintain the temperature of the knee joint below the body temperature, the findings of this study suggested that traditional cryotherapy using ice/gel pack could achieve a similar decrease in temperature and reach similar clinical effects. However, a significant weakness of these trials is that neither the skin temperature nor the intraarticular temperature was persistently measured and monitored to confirm effective cooling (17–22). Currently, the optimal cold treatment protocol remains unclear, including the cold temperature, application time and interval, and whether it needs relevant adjustment for different joints. Therefore, further exploration of the application of cryotherapy should be considered, and attention should also be paid that statistically significant findings may not translate into clinically significant results.

On the other hand, as healthcare providers, it is our duty to appropriately allocate finite resources to evidence-based approaches that are efficacious in an era of increasing expenses. Therefore, apart from the convenience that continuous cryotherapy devices provide prolonged continuous cooling and do not need to change the ice/gel pack, which does not offer any extra clinical advantage for patients undergoing TKA when compared with traditional cryotherapy (13). However, continuous cryotherapy warrants additional costs and resources associated with providing the cooling devices, which may not be covered by insurance (13, 17, 19, 22). In comparison, the cost of traditional cryotherapy is almost neglectable but achieves similar clinical effects. Thus, the currently available evidence does not support the theoretical cost-effectiveness of utilizing continuous cryotherapy after TKA, and future high-level prospective studies are needed to verify these findings.

In addition, the current available RCTs only applied continuous cryotherapy and traditional cryotherapy during hospitalization and not after discharge, which is a relatively short duration, and the minimal difference between continuous cryotherapy and traditional cryotherapy may not be detected (17, 29). Therefore, the extended application of cryotherapy at home could also be explored in future studies.

### 5.3 Strengths and limitations

To the best of our knowledge, this is the first systematic review and meta-analysis that systemically and comprehensively reviewed currently available evidence to compare the efficacy, safety, and cost-effectiveness of continuous cryotherapy vs. traditional cryotherapy for TKA.

Our study also has several potential limitations. First of all, in more than half of included studies, neither patients nor healthcare providers were blinded to group allocation and outcome assessment, hence, subjective assessments such as pain level are subject to potential bias. Second, the comparison of continuous cryotherapy vs. traditional cryotherapy was specialized to the TKA procedure, which may not be generalizable to other surgical procedures, such as arthroscopic surgery. Third, substantial heterogeneity across studies was noticed, which may be explained by the considerable difference in cryotherapy protocols. Lastly, almost all eligible trials included in the meta-analysis had relatively modest sample sizes (<100 patients), and overestimation of the treatment effect is more likely than in larger trials.

## 6. Conclusion

Our systematic review and meta-analysis suggested that continuous cryotherapy showed no superiority in reducing pain intensity, analgesics consumption, swelling, blood loss, length of hospital stay, and improving ROM compared with traditional cryotherapy in the acute postoperative setting after TKA. Continuous cryotherapy may further lead to extra costs and resources, so the currently available evidence does not support continuous cryotherapy could be added as an adjunct therapy. Additional well-designed studies with larger sample sizes are needed to confirm these preliminary results.

## Data Availability

The original contributions presented in the study are included in the article/Supplementary Material, further inquiries can be directed to the corresponding author.

## Appendix 1. Literature search strategy.

**Table:**

Database			
PubMed	Search	Query	Results
	#1	"Cryotherapy"[Mesh]	26,791
	#2	Cryotherapy[Title/Abstract]	8,064
	#3	Cryopneumatic[Title/Abstract]	4
	#4	Cryo*[Title/Abstract]	101,381
	#5	Cold[Title/Abstract]	132,678
	#6	Cold therapy[Title/Abstract]	359
	#7	Cold treatment[Title/Abstract]	1,157
	#8	Ice[Title/Abstract]	35,670
	#9	Ice Bag*[Title/Abstract]	146
	#10	Ice pack*[Title/Abstract]	619
	#11	Icing[Title/Abstract]	914
	#12	Cooling[Title/Abstract]	43,608
	#13	Cooling water[Title/Abstract]	562
	#14	Cold Effects[Title/Abstract]	1,096
	#15	#1 OR #2 OR #3 OR #4 OR #5 OR #6 OR #7 OR #8 OR #9 OR #10 OR #11 OR #12 OR #13 OR #14	310,602
	#16	"Arthroplasty, Replacement, Knee"[Mesh]	28,337
	#17	Knee Arthroplasty[Title/Abstract]	27,828
	#18	Knee Replacement[Title/Abstract]	9,811
	#19	#16 OR #17 OR #18	40,053
	#20	#15 AND #19	180
**Embase**	**Search**	**Query**	**Results**
	#1	“cryotherapy”/exp	38,217
	#2	0ôcryotherapy device”/exp	97
	#3	cryotherapy:ab,ti	11,183
	#4	cryopneumatic:ab,ti	4
	#5	cryo*:ab,ti	130,360
	#6	cold:ab,ti	165,147
	#7	“cold therapy”:ab,ti	273
	#8	“cold treatment”:ab,ti	1,064
	#9	“ice”:ab,ti	40,261
	#10	“ice bag*”:ab,ti	182
	#11	“ice pack*”:ab,ti	922
	#12	“icing”:ab,ti	904
	#13	cooling:ab,ti	46,673
	#14	“cooling water”:ab,ti	938
	#15	temperature:ab,ti	661,805
	#16	#1 OR #2 OR #3 OR #4 OR #5 OR #6 OR #7 OR #8 OR #9 OR #10 OR #11 OR #12 OR #13 OR #14 OR #15	972,974
	#17	“replacement arthroplasty”/exp	50,510
	#18	“hip arthroplasty”:ab,ti	28,377
	#19	“hip replacement”:ab,ti	15,115
	#20	“knee arthroplasty”:ab,ti	30,517
	#21	“knee replacement”:ab,ti	12,500
	#22	#17 OR #18 OR #19 OR #20 OR #21	92,662
	#23	#16 AND #22	895
	#24	#16 AND #22 AND [animals]/lim	63
	#25	#16 AND #22 AND [pubmed-not-medline]/lim	4
	#26	#16 AND #22 AND [erratum]/lim	5
	#27	#16 AND #22 AND ([animal cell]/lim OR [animal experiment]/lim OR [animal model]/lim OR [animal tissue]/lim)	34
	#28	#24 OR #25 OR #26 OR #27	69
	#29	#23 NOT #28	826
**Cochrane**	**Search**	**Query**	**Results**
	#1	MeSH descriptor: [Cryotherapy] explode all trees	1,683
	#2	(Cryopneumatic):ti,ab,kw (Word variations have been searched)	2
	#3	(Cryo*):ti,ab,kw (Word variations have been searched)	5,990
	#4	(Cold therapy):ti,ab,kw (Word variations have been searched)	4,323
	#5	(Cold treatment):ti,ab,kw (Word variations have been searched)	5,247
	#6	(Ice):ti,ab,kw (Word variations have been searched)	2,506
	#7	(Ice Bag*):ti,ab,kw (Word variations have been searched)	160
	#8	(Ice pack*):ti,ab,kw (Word variations have been searched)	395
	#9	(Cold):ti,ab,kw (Word variations have been searched)	11,820
	#10	(Cryotherapy):ti,ab,kw (Word variations have been searched)	2,309
	#11	(Icing):ti,ab,kw (Word variations have been searched)	2,457
	#12	(Cooling):ti,ab,kw (Word variations have been searched)	4,568
	#13	(Cooling water):ti,ab,kw (Word variations have been searched)	678
	#14	(Temperature):ti,ab,kw (Word variations have been searched)	23,270
	#15	#1 or #2 or #3 or #4 or #5 or #6 or #7 or #8 or #9 or #10 or #11 or #12 or #13 or #14	40,683
	#16	MeSH descriptor: [Arthroplasty] explode all trees	5,389
	#17	(Knee Replacement):ti,ab,kw (Word variations have been searched)	6,062
	#18	(Hip Arthroplasty):ti,ab,kw (Word variations have been searched)	5,506
	#19	(Hip Replacement):ti,ab,kw (Word variations have been searched)	5,550
	#20	(Knee Arthroplasty):ti,ab,kw (Word variations have been searched)	7,703
	#21	#16 or #17 or #18 or #19 or #20	16,065
	#22	#15 and #21	381
**Web of Science**	**Search**	**Query**	**Results**
	#1	TS = (Cryotherapy)	9,906
	#2	TS = (Cryopneumatic)	5
	#3	TS = (Cryo*)	201,930
	#4	TS = (Cold)	463,731
	#5	TS = (Cold therapy)	8,786
	#6	TS = (Cold treatment)	51,183
	#7	TS = (Ice)	222,234
	#8	TS = (Ice Bag*)	554
	#9	TS = (Ice pack*)	6,152
	#10	TS = (Icing)	222,269
	#11	TS = (Cooling)	457,798
	#12	TS = (Cooling water)	88,667
	#13	TS = (Cold Effects)	124,148
	#14	#13 OR #12 OR #11 OR #10 OR #9 OR #8 OR #7 OR #6 OR #5 OR #4 OR #3 OR #2 OR #1	1,241,609
	#15	TS = (Knee Arthroplasty)	47,281
	#16	TS = (Knee Replacement)	33,322
	#17	#16 OR #15	57,951
	#18	#14 AND #17	338
			
**Google Scholar**	**Search**	**Query**	**Results**
	#1	(“Cryotherapy” OR “Cryopneumatic” OR “Cold therapy” OR “Cold treatment” OR “Ice Bag” OR “Ice pack” OR “Cooling water”) AND (“total knee replacement” OR “total knee Arthroplasty”) AND (“Randomized Controlled Trial” OR “Controlled Clinical Trial”)	1,590
